# Postprandial lipemia in men with metabolic syndrome, hypertensives and healthy subjects

**DOI:** 10.1186/1476-511X-4-21

**Published:** 2005-09-30

**Authors:** Genovefa D Kolovou, Katherine K Anagnostopoulou, Antonis N Pavlidis, Klelia D Salpea, Stella A Iraklianou, Konstantinos Tsarpalis, Dimitris S Damaskos, Athanasios Manolis, Dennis V Cokkinos

**Affiliations:** 1 Cardiology Department, Onassis Cardiac Surgery Centre, Athens, Greece; 2 Molecular Biology Department, Onassis Cardiac Surgery Centre, Athens, Greece; 3 Medical Department, Tzanio State Hospital, Piraeus, Greece; 4 Boston University, School of Medicine, Boston, USA

## Abstract

**Background:**

The metabolic syndrome (MetS), as well as postprandial hypertriglyceridemia, is associated with coronary heart disease. This study aimed to evaluate the postprandial lipemia after oral fat tolerance test (OFTT) in subjects with MetS and compare them to hypertensive (HTN) and healthy subjects.

**Results:**

OFTT was given to 33 men with MetS (defined by the Adult Treatment Panel III), 17 HTN and 14 healthy men. The MetS group was further divided according to fasting triglycerides (TG) into TG ≥ 150 [MetS+TG, (n = 22)] or <150 mg/dl [MetS-TG (n = 11)], and into those with or without hypertension [MetS+HTN (n = 24), MetS-HTN (n = 9), respectively]. TG concentrations were measured before and at 4, 6 and 8 h after OFTT and the postprandial response was quantified using the area under the curve (AUC) for TG.

The postprandial response was significantly higher in MetS compared to HTN and healthy men [AUC (SD) in mg/dl/h; 2534 ± 1016 vs. 1620 ± 494 and 1019 ± 280, respectively, p ≤ 0.001]. The TG levels were increased significantly in MetS+TG compared to MetS-TG subjects at 4 (p = 0.022), 6 (p < 0.001) and 8 hours (p < 0.001). The TG were increased significantly in MetS-TG compared to healthy subjects at 4 (p = 0.011), 6 (p = 0.001) and 8 hours (p = 0.015). In linear regression analysis only fasting TG levels were a significant predictor of the AUC (Coefficient B = 8.462, p < 0.001).

**Conclusion:**

Fasting TG concentration is the main determinant of postprandial lipemia. However, an exaggeration of TG postprandialy was found in normotriglyceridemic MetS and HTN compared to healthy subjects. This suggests that intervention to lower fasting TG levels should be recommended in MetS subjects.

## Background

In 1988, Reaven et al. proposed insulin resistance as the underlying metabolic aberration linking essential hypertension (HTN), dyslipidemia, type 2 diabetes and other abnormalities associated with an increased risk of atherosclerotic cardiovascular disease in adults [1]. This constellation is now designated as the metabolic syndrome (Mets). Since then, many epidemiological studies have shown that MetS is associated with an increased incidence of coronary heart disease (CHD) [2,3]. MetS is clinically characterized by the presence of abnormal fasting triglyceride (TG) levels, low high-density lipoprotein (HDL) cholesterol levels, elevated plasma glucose, HTN and abdominal obesity. However, according to the National Cholesterol Education Program – Adults Treatment Panel (ATP III) guidelines, the diagnosis of MetS is present when at least three of the above five clinical criteria co-exist [4]. Although estimates of the prevalence are critically dependent on the exact definition used, the MetS has reached epidemic proportions. The age-adjusted prevalence of the MetS in the United States is estimated at 24% and increases to 44% in adults who are over 60 years old [5]. Similar results were found in Greek population (19.8%) [6].

Postprandial hypertriglyceridemia is also associated with cardiovascular disease [7]. During the postprandial state, TG rich lipoproteins such as chylomicrons, very low-density lipoproteins and their remnants, may promote the development of atherogenic small dense low-density lipoprotein (LDL) particles [8]. Abnormal postprandial lipemia has been observed in normolipidemic men with or without CHD [9], in heterozygous familial hypercholesterolemia (hFH), in women with CHD, in hypertensives and others, according to previous studies including ours [10-14]. Only few data exist regarding the response of TG to fatty meal in subjects with MetS [15]. The aim of this study was to evaluate the postprandial lipemia after an oral fat tolerance test (OFTT) in patients with MetS (defined by ATP III).

## Results

All participants ingested their fatty meal and tolerated it well. The amount of fatty meal ingested by the MetS group was 362(28) g, by the HTN was 333(23) g, and by the Healthy was 347(16) g.

### Clinical Characteristics

Clinical characteristics of main groups (MetS, HTN, and Healthy) are shown in Table 1. Clinical characteristics of subgroups (MetS+HTN, MetS-HTN, MetS+TG and MetS-TG) are shown in Table 2.

**Table 1:** Clinical characteristics of the 3 main study groups.

Characteristics	MetS n = 33	HTN n = 17	Healthy n = 14	P values
Age (years)	50(11)	52(10)	49(9)	0.632
BMI (kg/m^2^)	30(4)	26(2)	26(2)	<0.001
Waist (cm)	107(11)	93(5)	95(6)	<0.001
CHD -/+	25/8	17/0	14/0	
Hypertension -/+	9/24	0/17	14/0	
Systolic BP (mmHg)	141(19)	149(18)	121(7)	<0.001
Diastolic BP (mmHg)	83(12)	92(11)	74(9)	<0.001
Smokers -/+	12/21	9/8	14/0	
Diabetes mellitus -/+	26/7	16/1	14/0	
TC (mg/dl)	241(39)	214(29)	188(28)	<0.001
HDL (mg/dl)	35(7)	48(15)	51(15)	<0.001
LDL (mg/dl)	165(36)	145(31)	113(33)	<0.001
Apo A (mg/dl)	133(19)	148(30)	159(48)	0.038
Apo B (mg/dl)	137(28)	124(23)	125(29)	0.232
Lp (a) (mg/dl)	16(13)	21(16)	16(11)	0.57
Glucose (mg/dl)	109(29)	93(10)	91(10)	0.013
Insulin (μU/ml)	14(9)	8(9)	7(5)	0.036
HOMA-IR	3.9(2.7)	2.1(2.2)	1.7(1.1)	0.015
TG0 (mg/dl)	204(86)	105(30)	89(30)	<0.001
TG4 (mg/dl)	364(153)	242(84)	145(44)	<0.001
TG6 (mg/dl)	366(155)	269(94)	142(53)	<0.001
TG8 (mg/dl)	297(124)	200(87)	131(56)	<0.001
AUC (mg/dl/h)	2534(1016)	1620(494)	1019(280)	<0.001

All values are presented as means (standard deviation) or percentages for categorical variables. P values arose by ANOVA analysis and denote difference among the three groups. MetS: men with metabolic syndrome, HTN: Hypertensive men, BMI: body mass index, CHD: coronary heart disease, TC: total cholesterol, HDL: high-density lipoprotein cholesterol, LDL: low-density lipoprotein cholesterol, Apo: apolipoprotein, Lp: lipoprotein, TG0: fasting plasma triglyceride concentration, TG4, TG6, TG8: plasma triglyceride concentration 4, 6, 8 h after the fat load, respectively, HOMA-IR: index of homeostasis model of insulin resistance, AUC: area under the curve for triglycerides. To convert from mg/dl to mmol/L for TC, HDL, LDL, divide by 38.7. To convert from mg/dl to mmol/L for glucose, divide by 18. To convert from mg/dl to mmol/L for TG divide by 88.6.

**Table 2:** Clinical characteristics of different subgroups.

Characteristics	MetS+HTN	MetS-HTN	MetS-TG	MetS+TG
Number of patients	24	9	11	22
Age (years)	53(10)	40(6)	52(10)	48(11)
BMI (kg/m^2^)	30(4)	31(3)	30(4)	30(4)
Waist (cm)	105(10)	115(10)	104(9)	108(11)
CHD -/+	16/8	9/0	6/5	19/3
Hypertension -/+	0/24	9/0	1/10	8/14
Systolic BP (mmHg)	148(17)	123(11)	143(12)	140(22)
Diastolic BP (mm Hg)	85(13)	78(6)	82(15)	83(11)
Smokers -/+	10/14	2/7	4/7	8/14
Diabetes mellitus -/+	17/7	9/0	7/4	19/3
TC (mg/dl)	234(37)	259(40)	228(34)	247(40)
HDL (mg/dl)	36(7)	30(3)	34(6)	35(7)
LDL (mg/dl)	159(34)	179(38)	166(33)	164(38)
Apo A (mg/dl)	137(19)	124(17)	130(10)	135(23)
Apo B (mg/dl)	135(29)	143(25)	134(36)	139(24)
Lp (a) (mg/dl)	16(11)	17(19)	16(10)	17(15)
Glucose (mg/dl)	113(33)	101(11)	114(35)	107(26)
Insulin (mg/dl)	12(6)	22(14)	14(9)	15(10)
HOMA	3.4(2.1)	5.8(3.9)	3.7(2.4)	4.1(2.9)
TG0 (mg/dl)	187(86)	248(72)	125(22)	243(78)
TG4 (mg/dl)	327(116)	454(199)	232(96)	411(143)
TG6 (mg/dl)	344(150)	426(161)	256(95)	421(151)
TG8 (mg/dl)	287(126)	321 (121)	200(73)	343(117)
AUC (mg/dl/h)	2349(945)	2984(1099)	1639(549)	2960(907)

All values are presented as means (standard deviation) or percentages for categorical variables. MetS: men with metabolic syndrome, HTN: Hypertensive men, BMI: body mass index, CHD: coronary heart disease, TC: total cholesterol, HDL: high-density lipoprotein cholesterol, LDL: low-density lipoprotein cholesterol, Apo: apolipoprotein, Lp: lipoprotein, TG0: fasting plasma triglyceride concentration, TG4, TG6, TG8: plasma triglyceride concentration 4, 6, 8 h after the fat load, respectively, HOMA-IR: index of homeostasis model of insulin resistance, AUC: area under the curve for triglycerides. To convert from mg/dl to mmol/L for TC, HDL, LDL, divide by 38.7. To convert from mg/dl to mmol/L for glucose, divide by 18. To convert from mg/dl to mmol/L for TG divide by 88.6.

### Postprandial Changes

Plasma total and HDL cholesterol, apolipoproteins A and B, and lipoprotein (a) were measured postprandialy in a 60% of the study population and no significant difference was found between groups.

**Figure 1:**
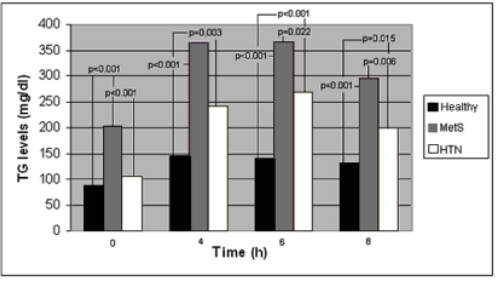
Comparison of triglyceride (TG) concentrations at 0, 4, 6, 8 h between pairs of the three main groups [men with metabolic syndrome (MetS), Hypertensive men (HTN) and Healthy].

**Figure 2:**
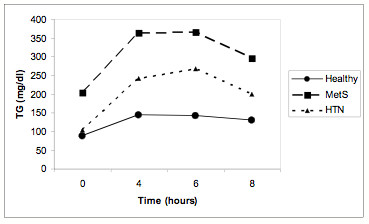
**Schematic representation of postprandial response (area under the curve) of the three main groups [men with metabolic syndrome (MetS), Hypertensive men (HTN) and Healthy]**. p = 0.001 for differences between MetS and HTN groups. p < 0.001 for differences between MetS and Healthy groups. p < 0.001 for differences between HTN and Healthy groups.

The following changes were noticed pre and post OFTT:

A) Comparison of the TG concentrations at 0, 4, 6, 8 h in MetS, HTN and Healthy subject groups are shown in Figure 1. Schematic representation of postprandial response (AUC) of the above three groups is shown in Figure 2.

B) In MetS+HTN and HTN groups: The TG levels were significantly increased in MetS+HTN vs. HTN subjects at 4 hours (p = 0.022), 6 (p < 0.001) and 8 hours (p < 0.001). [Mean (SD) values are presented in Table 1, 2].

C) In MetS+TG and MetS-TG: The TG levels were increased significantly in MetS+TG vs. MetS-TG subjects at 4 (p = 0.022), 6 (p < 0.001) and 8 hours (p < 0.001). [Mean (SD) values are presented in Table 2].

D) In MetS-TG and Healthy: The TG levels were increased significantly in MetS-TG vs. Healthy subjects at 4 (p = 0.011), 6 (p = 0.001) and 8 hours (p = 0.015). [Mean (SD) values are presented in Table 1, 2].

E) No significant differences were found in MetS+HTN vs. MetS-HTN and in MetS-TG vs. HTN subjects.

F) In linear regression analysis, where the AUC was the dependent variable and age, body mass index (BMI), HDL cholesterol, fasting TG, fasting glucose and HOMA-IR were the independent variables, only fasting TG levels were independently associated with high AUC values (Coefficient B = 8.462, p < 0.001).

## Discussion

In the present study we investigated the effect of a fatty meal on plasma TG concentration in untreated men with MetS, HTN and Healthy. We showed that MetS men have a different response to a fatty meal when compared to HTN and Healthy men. Linear regression analysis showed that for every rise of 1 mg/dl of the fasting TG values, the AUC increased by 8.462 mg/dl/h.

The fasting TG values were 129% higher in MetS compared to Healthy men. The observed difference was smaller than that reported by Chan et al [16]. Several facts could explain this difference: First, the fasting TG levels in their control group were much lower compared to our group. Fasting TG levels of their MetS subjects were also lower even though the BMI was higher. Indeed, when the clinical characteristics were similar to those of our subjects, the fasting TG values did not differ substantially [17]. Furthermore, the regulation of plasma TG in MetS men may differ according to concomitant characteristics. An enhanced TG rise postprandialy has been reported not only in patients with CHD [18], but also in patients with diabetes mellitus [19] and obesity [20], all of which are common findings in MetS subjects.

Obesity is associated with a range of metabolic abnormalities including fasting and postprandial dyslipidemia. This point is considered in greater detail; see our review [21]. In summary, visceral adiposity with increased intra-abdominal fat has been shown to precede the development of insulin resistance. The insulin resistance is associated with dyslipidemia [22] and may affect chylomicron remnant metabolism by down regulating LDL-receptor expression and increasing hepatic cholesterol synthesis and very low-density lipoprotein secretion [23]. These effects increase competition between chylomicron and very low density lipoprotein remnants for hepatic receptors, thereby impairing the uptake of chylomicron remnants by this pathway [24]. These changes result in elevated postprandial TG levels. Obesity has similar potential effects on chylomicron remnant metabolism. However, a weight reduction of 10 kg in insulin-resistant obese men was insufficient to reduce the elevated chylomicron remnant levels [25]. In contrast, type 2 diabetic patients treated with insulin, showed a 28% decrease in fasting plasma TG levels, a 17% increase in total HDL cholesterol, while the magnitude of postprandial lipemia after the ingestion of fatty meal decreased by 38%. Additionally, insulin treatment was accompanied by a 21% increase in lipoprotein lipase activity and a 20% increase in cholesteryl ester transport protein activity [26].

Essential hypertension constitutes one of the features included in the MetS and is further associated with insulin resistance. We have previously reported [13] that hypertension is associated with an abnormal response to a fatty meal (increased TG levels and delayed TG clearance), which seems to aggravate the prognosis of hypertensive patients. In our current subjects with MetS and hypertension (24 out of 33 MetS subjects) the exaggerated postprandial TG response to a fatty meal was even more pronounced than in 17 subjects with hypertension as a single abnormality [at 4 hours (p = 0.022), 6 (p < 0.001) and 8 hours (p < 0.001)]. It is difficult to distinguish which mechanism is responsible for this. The variability of BMI or fasting TG levels between subjects with MetS and hypertension compared to subjects with hypertension only [30(4) vs. 26(2), or 187(86) vs. 105(30), respectively] can probably account for these differences. However, other mechanisms may also be responsible.

In our current study HDL cholesterol levels were found to be lower in the MetS subjects compared to Healthy men as expected. However, other studies, including ours [27], have suggested that the levels of HDL cholesterol do not affect postprandial TG magnitude. Specifically, in a subgroup of subjects with isolated low fasting HDL cholesterol, fasting hypertriglyceridemia was a prerequisite in order to have an exaggerated postprandial TG response [28]. Additionally, Cohen and co-workers [29] measured the response of plasma TG and retinyl palmitate to different fatty meals in endurance-trained men with a wide range of plasma HDL cholesterol concentrations (36–105 mg/dl). Their data indicated that the magnitude of postprandial lipemia is not primarily affected by HDL cholesterol concentration.

The remnants of TG-rich lipoproteins accumulated in the postprandial state are involved in atherogenesis. They act as carriers of cholesteryl ester to the vessel wall and also induce endothelial dysfunction with their toxic influence on endothelial cells [30]. Furthermore, the hypertriglyceridemic state is accompanied by small dense LDL particles that are more susceptible to oxidation and low concentrations of HDL cholesterol [31]. Hypertriglyceridemia may also be involved in events leading to thrombosis [32] and probably provoke activation of nuclear factor kappa B, a proposed key mediator of atherosclerosis [33]. In subjects with MetS, the hypertriglyceridemia is one of the main lipid abnormalities, which potentially is responsible for exaggerated TG postprandialy. However, in subjects with MetS and normal fasting TG [125(22) mg/dl], defined by ATP III guidelines (< 150 mg/dl), the TG levels postprandialy were exaggerated and delayed clearance was also observed. Besides, the postprandial response was similar in MetS subjects with hypertension compared to MetS subjects with normal blood pressure, even though the baseline TG levels were lower in the former [187(86) vs. 248(72) mg/dl, p = 0.041, respectively]. This suggests that after certain fasting TG levels, no further increase of postprandial TG occurs; alternatively other mechanisms could be involved such as genetic influence.

## Conclusion

Our data indicate that fasting TG concentration is the main determinant of postprandial lipemia. However, an exaggeration of TG postprandialy is observed in MetS and HTN subjects with normal fasting TG levels. This suggests that intervention to lower fasting TG levels should be recommended in MetS subjects.

## Methods

### Study population

The study population consisted of 64 Greek men. Heavy drinking, liver and renal disease, hypothyroidism, professional sport activity and the use of hypolipidemic drugs were exclusion criteria. The study population was divided into 3 groups:

1. The MetS group consisted of 33 men, mean age 50 ± 11 years. The diagnosis of MetS was based on the co-existence of at least three of the following five clinical criteria: a) abdominal obesity (waist >102 cm), b) serum TG levels ≥ 150 mg/dl, c) serum HDL cholesterol levels <40 mg/dl, d) blood pressure ≥ 130/85 mmHg, e) fasting glucose >110 mg/dl.

2. The Hypertensive (HTN) group consisted of 17 men, mean age 52 ± 10 years with a negative CHD history. Additionally, their serum TG levels were < 150 mg/dl, and none of them fulfilled the criteria for MetS.

3. The Healthy group consisted of 14 healthy men, age-matched, mean age 49 ± 9 years with no family history of premature atherosclerosis, diabetes mellitus, HTN and dyslipidemia. Their fasting TG levels were < 150 mg/dl, their fasting glucose < 110 mg/dl, their total cholesterol < 240 mg/dl, their LDL <160 mg/dl, and their blood pressure < 130/85 mmHg. All healthy subjects were never smokers.

The MetS group was further divided into those with baseline TG levels < 150 mg/dl (MetS-TG, n = 11) and into those with TG levels ≥ 150 mg/dl (MetS+TG, n = 22), and into MetS with and without HTN [MetS+HTN (n = 24) and MetS-HTN (n = 9), respectively].

All participants gave their informed consent and the Ethics Committee of the Onassis Cardiac Surgery Centre, Athens, Greece, approved the study protocol.

### Study Protocol

All patients were studied in the outpatient clinic between 8.00–9.00 am after 12 h overnight fast. The fatty meal was consumed within 20 min and plasma TG concentrations were measured before and 4, 6 and 8 h after the fat load. During this 8 h period, the participants did not eat and did not smoke. They were only allowed to drink water. Blood samples were drawn at 8:00 (before the meal), at 12:30 am (4 h after the meal), at 2:30 pm (6 h after the meal), and at 4:30 pm (8 h after the meal). In all four samples total cholesterol, TG, HDL cholesterol, apolipoproteins A and B, and lipoprotein (a) were measured. The content of the fatty meal has been described in a previous study [13]. Briefly, the fatty meal was a slight modification of that introduced by Patsch et al. [9], consisting of 83.5% fat, 14.0% carbohydrates and 2.5% proteins and was given in a dose based on the patient's body surface area (350 g for 2 m^2^).

### Determination of blood lipids and glucose

Plasma total cholesterol, TG and HDL cholesterol were measured using enzymatic colorimetric methods on a Roche Integra Biochemical analyzer with commercially available kits (Roche Diagnostics Gmbh, Hannheim, Germany). The serum LDL cholesterol levels were calculated using the Friedewald formula [34] only in patients with TG levels <400 mg/dl. Apolipoproteins A, B and lipoprotein (a) were measured by nephelometery (Nephelometer: BN-100, Behring, Germany). Blood glucose was measured by the hexokinase method with a Dade Behring reagent on a Dimension (Dade Behring) instrument. Blood insulin was measured with the IMX ABBOTT Diagnostics instrument. All samples were analyzed within 24 h.

The body mass index was calculated as weight divided by height expressed in kg/m2. We assessed the whole-body insulin resistance with the following formula: HOMA-IR = (fast glucose × fast insulin)/22.5 [13].

### Statistical Analysis

Categorical variables are presented as percentages. Values of numerical characteristics were tested for normality and are presented as mean value (± SD), if normally distributed. An ANOVA or Kruskal-Wallis H test with a Bonferroni correction (whichever appropriate) was performed for three group comparisons. The t-test for independent samples or the Mann Whitney U test was used for the comparison of numerical values between two groups. Areas under the curve (AUC) for serial measurements of TG levels at baseline and after the fatty meal were calculated using the trapezoid rule. Linear regression analysis, where the AUC was the dependent variable and age, BMI, HDL cholesterol, fasting TG, fasting glucose and HOMA-IR were the independent variables was performed in order to uncover the significant predictors of postprandial lipemia (i.e. high AUC values). The level of significance was set at p < 0.05.

## Authors' contributions

GDK conceived the study and participated in the development of the hypothesis, the study design and drafting of the manuscript. KKA is a research associate who participated in the development of the hypothesis, the study design and drafting of the manuscript. ANP is a physician who participated in the study design, recruitment of subjects and clinical evaluation. KDS is a research associate who participated in data analysis and interpretation of the findings. SAI, KT and DSD are physicians who participated in the study design, recruitment of subjects and clinical evaluation. AM is a physician who participated in the study design and drafting of the manuscript. DVC is a physician who participated in the design of the study and its coordination.
